# Prevalence of HBV DNA among 20 million seronegative blood donations in China from 2010 to 2015

**DOI:** 10.1038/srep36464

**Published:** 2016-11-11

**Authors:** Chao Liu, Le Chang, Huimin Ji, Fei Guo, Kuo Zhang, Guigao Lin, Rui Zhang, Jinming Li, Lunan Wang

**Affiliations:** 1 National Center for Clinical Laboratories, Beijing Hospital, National Center of Gerontology, Beijing, People’s Republic of China; 2 Beijing Engineering Research Center of Laboratory Medicine, Beijing Hospital, Beijing, People’s Republic of China; 3 Graduate School, Peking Union Medical College, Chinese Academy of Medical Sciences, Beijing, People’s Republic of China

## Abstract

The prevalence of HBV DNA among seronegative blood donations in China has not been studied extensively on a nationwide scale. The aim of this study was to analyse the prevalence, trend, distributions, and serological characteristics of HBV DNA positive/seronegative blood donations. We collected HBV test data from all blood banks of China from 2010 to 2015 and performed supplemental serological tests and quantitative detection of HBV DNA of the seronegative/HBV DNA positive blood donations. We analysed the prevalence of HBV DNA among seronegative blood donations screened by varying nucleotide acid test (NAT) reagents. The analysis results showed that a total of 20,084,187 seronegative blood donations were screened by NAT from 2010 to 2015 in China. The average frequency of HBV DNA among seronegative blood donations was 1/1482, but there has been a steady increase from 1/1861 in 2011 to 1/1269 in 2015. The geographical distribution of seronegative and HBV DNA positive blood donations was roughly consistent with that of HBsAg. The most common serological pattern was HBeAb and HBcAb positive. In conclusion, our study offeres fundamental data of seronegative and HBV DNA positive blood donations throughout China.

Hepatitis B virus (HBV) infection remains a significant public health concern worldwide, with more than 350 million chronic HBV carriers around the world^1^. Roughly, 786,000 people die of HBV-related diseases every year and HBV infection is the tenth leading cause of death^2^. China is a highly endemic area of HBV infection with one-third of the 350 million HBV carriers living in China^3^. According to epidemiological surveys, 9.8% of the Chinese population were HBsAg positive in 1992. With the implement of mandatory HBV vaccination among children, the HBsAg positivity rate drop to 7.2% in 2006^4^. Blood transfusion is one potential ways in which HBV transmission occurs between people. The risk of transfusion transmitted HBV (TTHBV) is a challenge for blood safety in China. Due to the high prevalence of HBV in China, blood donors are initially screened by an HBsAg rapid test before donation, and then the donated blood is tested using two different HBsAg ELISA kits. However, there is still a risk of TTHBV infection via blood donated by HBsAg negative donors who are in the window period (WP) or chronic occult HBV infection (OBI) stage^5^,^6^. Pre-seroconversion WP donations are likely to transmit HBV, but the transmission rate of OBI is low. Studies have also suggested that the risk of TTHBV infection is lower in highly endemic areas compared to that in non-endemic areas, because most recipients had experienced HBV infection^7^. Antibody screening for the Hepatitis B core antigen (anti-HBc) screening was implemented to eliminate OBI transmission risk in countries with low prevalence of HBV; however, it could not eliminate WP transmission risk. Nucleic acid test (NAT) analysis of blood donors has been reported to detect potential infectious HBV in the WP and OBI stages, which provides additional safety to blood transfusion^8^. In 2010, the Ministry of Health of the People’s Republic of China started a NAT screening program for HBV, hepatitis C virus, and human immunodeficiency virus infection in several blood banks. From 2010 to 2015, many blood banks began to implement NAT screening. The prevalence of HBV DNA among seronegative blood donations has been reported in other countries and several blood banks of China^9^,^10^,^11^. However, to date, no study has reported on the frequency of HBV DNA positivity among seronegative blood donors throughout China.

This study is aimed to: (1) calculate the prevalence of HBV DNA positive/ seronegative blood donors, (2) evaluate the trends in the prevalence of HBV DNA positive/seronegative blood donors over the last six years, (3) analyse the geographical distribution of the prevalence within the country, (4) calculate the percentage of total detection number and prevalence of HBV DNA positive/seronegative of NAT reagents, and (5) assess the serological characteristics of HBV DNA positive/seronegative blood donations.

## Results

### Number of NAT blood donations from 2010 to 2015 in China

**Figure 1:**
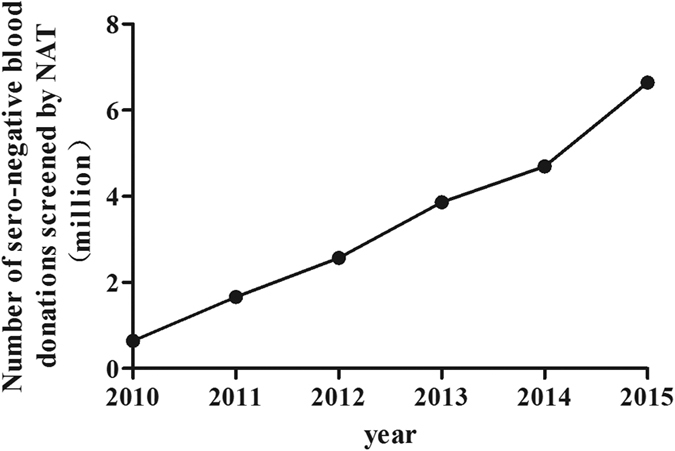
Trends in the total number of seronegative blood donations screened by NAT from 2010 to 2015.

From 2010 to 2015, a total of 20,084,187 seronegative blood donations were admitted for blood NAT screening in blood banks in China. Over the six-year period, the number of NAT blood donations increased steadily. The number was 642,028 in 2010; 1,665,968 in 2011; 2,567,975 in 2012; 3,865,045 in 2013; 4,696,705 in 2014; and 6,646,466 in 2015. (Fig. 1).

### Prevalence of HBV DNA positive/seronegative blood donations

**Figure 2:**
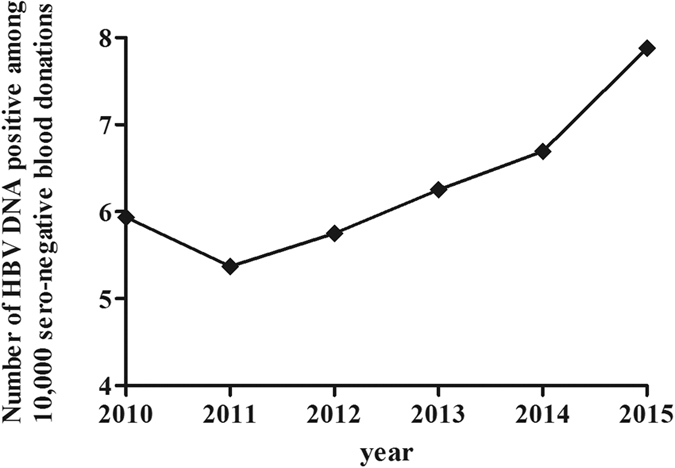
Prevalence of HBV DNA positivity among seronegative blood donations from 2010 to 2015. From 2010 to 2015, the prevalence of HBV DNA positivity among 10,000 seronegative blood donations was 5.93, 5.37, 5.75, 6.25, 6.69, and 7.88, respectively.

Among the 20,084,187 seronegative blood donations screened by NATs from 2010 to 2015, a total of 13,551 blood donations were HBV DNA positive, meaning that the average prevalence of HBV DNA was roughly 6.75 among 10,000 seronegative blood donations (1/1482). While the prevalence of HBV DNA positive/seronegative blood donations was different in each year, the prevalence in 2011 (5.37 among 10,000 seronegative) was lower than that of 2010 (5.93); the prevalence increased over the next five years. The prevalence from 2011 to 2015 was 5.37, 5.75, 6.25, 6.69, and 7.88 among 10,000 seronegative blood donations, respectively. (Fig. 2).

### Prevalence of HBV DNA positive/seronegative blood donors in different regions of China

**Figure 3:**
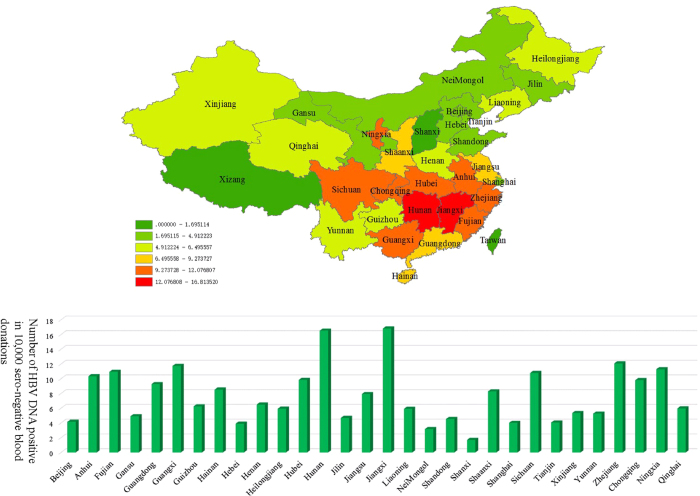
Geographical distribution of the prevalence of HBV DNA positive/seronegative blood donations (map was created by the ArcGIS software version 10 system http://www.esri.com/software/arcgis/). The prevalence varied geographically, and prevalence in the south was higher than that in the north. Jiangxi, Hunan, Zhejiang, Guangxi, and Ningxia ranked the top five provinces or province-level municipalities with the highest prevalence.

Among the 6,646,466 seronegative blood donations screened by NATs in 2015, 5237 were HBV DNA positive (7.88 per 10000). The data include all the provinces and province-level municipalities in the mainland of China with the exception of Xizang. The prevalence of HBV DNA positive/seronegative blood donations varied geographically. The areas with the highest prevalence of HBV DNA positive/seronegative blood donations were mostly located in the south of China. Jiangxi, Hunan, Zhejiang, Guangxi, and Ningxia ranked the top five provinces where the prevalence of HBV DNA among seronegative blood donations was the highest. The areas with the lowest prevalence were mostly located in the north of China (Fig. 3).

### Percentage of total detection number and prevalence of HBV DNA positive/seronegative by NAT reagents in China from 2010 to 2015

**Figure 4:**
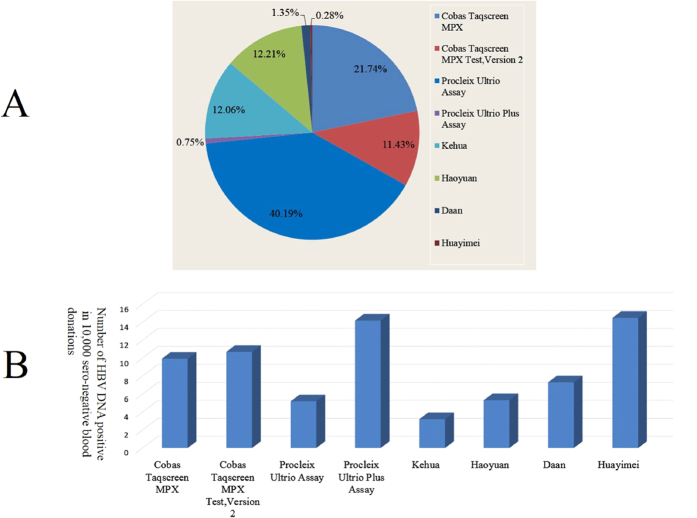
Percentage of total screened blood donations and prevalence of HBV DNA positivity among seronegative blood donations by each NAT reagent in blood screened from 2010 to 2015. (**A**) Percentage of total blood donations screened by each NAT reagent from 2010 to 2015. From highest to lowest: Procleix Ultrio Assay (40.19%); Cobas Taqscreen MPX (21.74%); Cobas Taqscreen MPX Test, Version 2 (11.43%); Haoyuan (12.21%); Kehua (12.06%); Daan (1.35%); Procleix Ultrio Plus Assay (0.75%); and Huayimei (0.28%). (**B**) Prevalence of HBV DNA positivity among 10,000 blood donations that were determined seronegative by NAT reagents. Prevalence by NAT reagent: Huayimei (14.48); Procleix Ultrio Plus Assay (14.17), Cobas Taqscreen MPX Test, Version 2 (10.69); Cobas Taqscreen MPX Test (9.93); Daan (7.3); Haoyuan (5.31); Procleix Ultrio Assay (5.19); and Kehua (3.21).

From 2010 to 2015, a total of eight different reagents were used for blood NAT screening; they can be divided into two groups: imported reagents including the Procleix Ultrio Plus Assay, Cobas Taqscreen MPX test Version 2, Procleix Ultrio Assay, Cobas Taqscreen MPX test, and domestic reagents including Daan, Huayimei, Haoyuan, and Kehua. Most (74.11%) of the blood donations were screened by imported reagents, among which the Procleix Ultrio Assay was predominantly used (40.19%). Among the domestic reagents, Haoyuan was used most (12.21%), and Huayimei was used the least (0.28%). We next calculated the prevalence of HBV DNA positive/seronegative blood donations by different reagents. The prevalence by Huayimei was the highest of all of the reagents (14.48 per 10,000); while the prevalence by Kehua was the lowest of all the reagents (3.21 per 10000). (Fig. 4).

### HBV serological characteristics of the seronegative and HBV DNA positive blood donations

A total of 4,601 seronegative and HBV DNA positive blood donations from blood banks around China were sent to the National Center for Clinical Laboratories (NCCL). Then, we assessed the serological pattern of all the blood donations. Among them, 10.34% were HBsAg positive or border. The most common patterns were HBeAb and HBcAb positive (21.06%), and HBcAb positive (20.04%). Among the HBsAg negative blood donations, the prevalence of HBcAb and HBeAg was 77.36% and 0.9%, respectively (Tables 1 and 2). In addition, there were also some uncommon serological patterns of HBV infection such as HBsAg negative and HBeAg positive blood donations.

### Quantitative detection of HBV in seronegative and HBV DNA positive blood donations

In this study, we performed quantitative detection of HBV in 3037 seronegative and HBV DNA positive blood donations. The concentration of HBV DNA ranged from undetectable to 34,600 IU/ml. Among them, 95.7% were below 20 IU/ml or undetectable. The HBV concentration of most of the seronegative and HBV DNA positive blood donations in China was low.

## Discussion

In 2010, the Ministry of Health of the People’s Republic of China started the NAT program to reduce the transmission risk of HBV, HCV, and HIV in blood donations. Seronegative blood donations were sent for NAT screening. The number of blood donations screened by NATs increased from 642,028 in 2010 to 6,646,466 in 2015. This was due to an increasing number of blood banks initiating NAT screening over the six-year period. At the same time, the number of seronegative blood donations that tested positive for HBV DNA increased from 381 in 2010 to 5237 in 2015. From 2010 to 2015, a total of 20,084,187 blood donations were screened by NAT, among which 13,551 were HBV DNA positive. The average prevalence of HBV DNA positive/seronegative blood donations was 1/1482 (6.75 per 10,000). In the areas with high endemicity of HBV, the prevalence of OBI in Hong Kong was roughly 1/3226, which was similar to the prevalence of 1/3239 in Shenzhen city of China, which is geographically close to Hong Kong^9^,^12^. In Taiwan, the prevalence was 1/909, which was similar to the prevalence of the neighbouring Fujian province^7^. Meanwhile, in the non-endemic areas, the prevalence was low. It was reported that the prevalence of HBV DNA positive/seronegative blood donations in Scotland and Poland was 1/1,666,667 and 1/61,047, respectively^10^,^11^.

The annual prevalence of HBV DNA positive/seronegative blood donations varied from 2010 to 2015. The prevalence increased from 5.93 per 10,000 in 2010 to 7.88 per 10,000 in 2015, which is roughly a 33% increase. The prevalence decreased from 5.93 in 2010 to 5.37 in 2011. However, the difference is not significant, and this may be a normal fluctuation. The prevalence then increased linearly from 5.37 in 2011 to 7.88 in 2015. There may be two reasons for this observation. First, an increasing number of blood banks in regions with a high prevalence of HBV infection in China began NAT screening, which increased the average level of the whole country. The proportions of blood donations from regions with high prevalence of HBV infection from 2010 to 2015 were 55.88%, 52.28%, 52.07%, 51.56%, 52.8%, and 59.31%, respectively. The varying proportions of blood donations from regions with high prevalence of HBV infection may contribute to the difference in prevalence of HBV DNA among seronegative blood donations from 2010 to 2015. Second, the prevalence was easily affected by the sensitivity of the HBsAg ELISA reagents and NAT kits. Newer NAT reagents with high sensitivity such as the Procleix Ultrio Plus Assay, Cobas Taqscreen MPX test Version 2, and Huayimei assay were used. Blood banks choose to use the high-sensitivity NAT kits.

To assess the geographical distribution of the prevalence of HBV DNA positive/seronegative blood donations across the country, we evaluated the prevalence of all of the provinces and province-level municipalities in the mainland of China with the exception of Xizang. Among them, the number of blood donations screened by NAT ranged from 18,417 in Qinghai to 636,460 in Shandong. The number of HBV DNA positive blood donations ranged from 11 in Qinghai to 568 in Zhejiang. The highest prevalence was 16.8 per 10,000 seronegative blood donations in Jiangxi, while the lowest was 1.8 per 10,000 in Shanxi. Among the 13 provinces with prevalence higher than 8 per 10,000, Ningxia and Shannxi were located in the North of China, while all of the other eleven provinces were located in the South of China. Most of the regions with relatively high prevalence were located in the South of China. This is consistent with the results of an HBsAg survey in 1992 showing that the prevalence of HBsAg in the north of China was lower than that of the south^13^. In regions with a high prevalence of HBsAg, most infections are contracted perinatally or in early childhood, and a higher proportion of the adult HBV infections would be in the late chronic occult HBV infection (OBI) stage^14^. The “healthy” HBsAg-negative individuals are potential blood donors, which would lead to the higher prevalence of HBV DNA among seronegative blood donations.

Because NAT reagents play an important role in the results of NAT blood screening, we also assessed the percentage of total detection number and prevalence of HBV DNA positive/seronegative blood donations by each reagent. From 2010 to 2015, 74.11% of the seronegative blood donations were screened by imported NAT reagents, including Cobas and Procleix Ultrio Assays. The remaining was shared by four domestic reagents: Kehua, Haoyuan, Daan, and Huayimei. Using the current detection methods of each reagent including minipool and individual donation test, the prevalence of HBV DNA as determined by domestic reagents was lower than that determined by the Procleix Ultrio Plus Assay and Cobas Taqscreen MPX test Version 2. However, the prevalence by Huayimei was similar to that by the second-generation imported reagents. However, the number of blood donations screened by Huayimei was limited and this reagent needs further validation. In this study, we only analysed the prevalence as determined using this reagent and we did not assess any other aspects such as their false positivity rate. Even though the prevalence of the reagents, they were also affected by detection number and region.

To assess the serological characteristics of the HBV DNA positive and HBsAg negative blood donations, we tested for HBsAg, anti-HBs, HBeAg, anti-HBe, and anti-HBc in 4,601 of the 13,551 HBV DNA positive and HBsAg negative blood donations using electrochemiluminescence immunoassay (ECLIA) and chemiluminescent microparticle immunoassay (CMIA). The results demonstrated that 8.28% and 2.06% of the blood donations were HBsAg positive or border, respectively. This is because ECLIA and CMIA are more sensitive than the ELISA test used in blood banks. The most common serological pattern was HBeAb and HBcAb positive (21.06%), and HBcAb positive (20.04%). Broadly speaking, occult hepatitis B infection (OBI) is defined as the presence of detectable HBV DNA in the liver or serum without detectable HBsAg in serum^15^. After excluding the HBsAg positive or border portion, the prevalence of OBI among seronegative blood donors was about 6.05 per 10,000 (1/1652). The meta-analysis results by Liu *et al*. showed that the prevalence of OBI among 571,227 Chinese volunteer blood donors was 9.4 per 10,000 (1/1063)^16^. The prevalence in our study was more than 54-fold higher than the average prevalence in the international survey in 2008^17^. In Taiwan, the prevalence was 1/747, which was higher than that of our study^18^. The difference may be due to the difference in prevalence of HBV infection between them. Usually, the prevalence of OBI is high in areas with a high level of HBV endemicity^19^,^20^. The transmission rate of OBI from blood donations is low in humans, especially the anti-HBs positive OBI donations^21^. A study by Su *et al*. demonstrated that the transmission rate of OBI is lower in highly endemic areas than that in non-endemic areas, because most recipients had experienced HBV infection^7^. Among the seronegative blood donations in our study, the prevalence of anti-HBc and anti-HBs was 77.36% and 39.56%, respectively. This was similar to the previously reported prevalence of anti-HBc (77.3%) and anti-HBs (31.8%) in Shenzhen city of China. The 4,601 blood donations were collected from blood banks in 24 provinces of China, which could represent the whole serological status of 13,551 blood donations to some extent. However, the results in our study do not necessarily accurately represent the whole status of seronegative and HBV DNA positive blood donations.

There were also several uncommon serological patterns of HBV in our study. There were 28 HBsAg negative and HBeAg positive blood donations. Several factors could lead to the uncommon serological pattern of HBV. First, HBV S gene mutation could lead to HBsAg negativity; second, if the titre of HBsAg is too high, it could lead to the “Hook effect” and give rise to a false negative result.

The major limitation of the study was that we stuied on different populations using ELISA and NAT reagents with different sensitivities, which made it difficult to evaluate the true prevalence of HBV DNA among seronegative blood donations and compare the prevalence between different years and regions. The prevalence of HBV DNA among seronegative blood donations, which included window cases and HBV chronic donations, were easily affected by the endemic level of HBV in different regions, different reagents used in the studies, and different populations studied^20^. The prevalence of OBI could be different when HBsAg and HBV DNA reagents with different sensitivities were used in the same study population^20^,^22^. In China, blood banks could choose HBsAg and NAT reagents, which were approved by CFDA. It is hard to expect all blood banks to use the same reagents. Under ideal conditions, all blood banks in China would use the same HBsAg and NAT reagents that were most sensitive, following which the calculated prevalence would be closer to true prevalence. The prevalence in our study demonstrated the actual prevalence of HBV DNA among seronegative blood donations throughout China, when blood banks use different HBsAg and NAT reagents. The actual prevalence in our study is different from the true prevalence. There is a long way to go to be able to evaluate the true prevalence.

In summary, during the period of 2010 to 2015, the number of blood donations screened by NAT increased linearly. The prevalence of HBV DNA positivity among seronegative blood donations varied from 2010 to 2015. The average prevalence of the six years was 1/1482 (6.75 per 10,000). The prevalence also varied with geographical distribution, which was roughly consistent with that of HBsAg. The most common serological pattern of seronegative and HBV DNA positive blood donations was HBeAb and HBcAb positive. At the same time, 8.28% and 2.06% of the blood donations tested by ECLIA and CMIA were HBsAg positive or border, respectively. According to the generalized concept of OBI, the prevalence of OBI among seronegative blood donations was 6.05 per 10,000 (1/1652). In conclusion, our study offers fundamental data of seronegative and HBV DNA positive blood donations throughout China.

The prevalence of HBV DNA among seronegative blood donations of China was relatively high. Nucleotide acid tests (NAT) are essential to ensure the safety of blood transfusion in China. NAT screening should be carried out in all blood banks of China and other countries with high prevalence of HBV infection. The viral load of most of the HBV DNA positive/seronegative blood donations was low and close to the assay limit. Thus, more sensitive NAT reagents should be used in blood screening.

## Methods

### Study design

The data were obtained from a NAT program for blood donation screening launched by Chinese National Health from 2010. Seronegative blood donors were screened for HBV, HCV, and HIV by NAT in blood banks. We collected HBV test data from all blood banks that enrolled in the program every month from 2010 to 2015. A proportion of HBV DNA positive/seronegative blood donations were sent to the National Center of Clinical Laboratories (NCCL) for further testing for HBV surface antigens (HBsAg), antibodies to HBsAg (anti-HBs), HBV e antigen (HBeAg), antibodies to HBeAg (anti-HBe), and HBV core antibody (anti-HBc) by electrochemiluminescence immunoassay (ECLIA; Roche Molecular Systems;) or chemiluminescent microparticle immunoassay (CMIA, ARCHITECT assays). The study was approved by the Ethics Committee of the National Center for Clinical Laboratories. The methods in the study were in accordance with the guidelines of the Declaration of Helsinki. Written informed consent was obtained from all subjects participating in this research.

### Screening procedures

Most of the blood donors passed a pre-donation examination including a medical history questionnaire, rapid screening, and simple physical examination. Blood donors underwent a rapid test for HBsAg and alanine aminotransferase (ALT) levels. The increased ALT levels and HBsAg positive were deferred. The blood donations were screened for HBsAg, antibody to HIV1/2 (anti-HIV1/2), antibody to HCV (anti-HCV), and antibody to syphilis (anti-syphilis) at blood centres twice using two different ELISA reagents, respectively. Then, all the HBsAg, anti-HIV1/2, anti-HCV, and anti- syphilis negative donations were further tested by minipool or individual NAT testing. NAT reactive donations should be further tested with the discriminatory NAT assays or individual donation test. HBV DNA positive blood donations were aborted and were not used in transfusions. All the reagents were approved by the China Food and Drug Administration and tests were conducted according to the instructions of the manufacturer. The 95% HBV DNA limit of detection of each NAT reagent were listed in Table 3.

### Supplemental testing and statistical analysis

Due to the restrictive conditions, only a proportion of the HBV DNA positive donations were further tested for HBsAg, anti-HBs, HBeAg, anti-HBe, and anti-HBc using ECLIA. HBV DNA quantification was performed using Roche COBAS AmpliPrep/TaqMan HBV first- or second-generation tests in a proportion of NAT reactive blood donations. A computer spreadsheet was used for data analysis. We calculated the prevalence of HBV DNA positivity among seronegative donors. We used GraphPad Prism version 6.0 (GraphPad, USA) and ArcGIS version 10 system (Esri, USA) software to assess the trend and geographical distribution of the prevalence of HBV DNA positive/seronegative blood donations.

## Additional Information

**How to cite this article:** Liu, C. *et al*. Prevalence of HBV DNA among 20 million seronegative blood donations in China from 2010 to 2015. *Sci. Rep.* **6**, 36464; doi: 10.1038/srep36464 (2016).

**Publisher’s note:** Springer Nature remains neutral with regard to jurisdictional claims in published maps and institutional affiliations.

## Figures and Tables

**Table 1:** Serological characteristics of the HBV DNA positive and seronegative blood donations.

HBsAg	HBsAb	HBeAg	HBeAb	HBcAb	Number(percent)
−	−	−	−	−	587(12.76%)
−	+	−	−	−	347(7.54%)
−	+	−	−	+	744(16.17%)
−	+	−	+	+	528(11.48%)
−	+	+	−	+	13(0.28%)
−	−	−	−	+	922(20.04%)
−	−	−	+	+	969(21.06%)
−	−	+	−	+	15(0.33%)
+^*^	+ or −	+ or −	+ or −	+ or −	381(8.28%)
Border^*^	+ or −	+ or −	+ or −	+ or −	95(2.06%)

*The samples were HBsAg positive or border tested by electrochemiluminescence immunoassay (ECLIA) or chemiluminescent microparticle immunoassay (CMIA) while they were negative when tested by ELISA.

**Table 2:** Prevalence of serological markers in HBsAg negative blood donations with HBV DNA positivity.

Serologic markers	Total
HBsAb	1632(39.56%)
HBeAg	28(0.68%)
HBeAb	1505(36.48%)
HBcAb	3191(77.36%)
Total	4125

**Table 3:** 95% HBV DNA limit of detection of each blood NAT screening reagent.

NAT screening reagents	Limit of detection (LOD)
Cobas TaqScreen MPX	3.7 IU/mL
Cobas Taqscreen MPX test Version 2	2.3 IU/mL
Procleix Ultrio Assay	10.4 IU/mL
Procleix Ultrio Plus Assay	3.4 IU/mL
Haoyuan	6.3 IU/mL
Huayimei	4.2 IU/mL
Kehua	6.2 IU/mL
Daan	≤100 IU/mL
